# Particle-Breaking Unrestricted Hartree–Fock Theory for Open Molecular Systems

**DOI:** 10.1021/acs.jpca.3c07231

**Published:** 2024-02-14

**Authors:** Regina Paul née Matveeva, Sarai Dery Folkestad, Bendik Støa Sannes, Ida-Marie Høyvik

**Affiliations:** Department of Chemistry, The Norwegian University of Science and Technology, Trondheim 7491, Norway

## Abstract

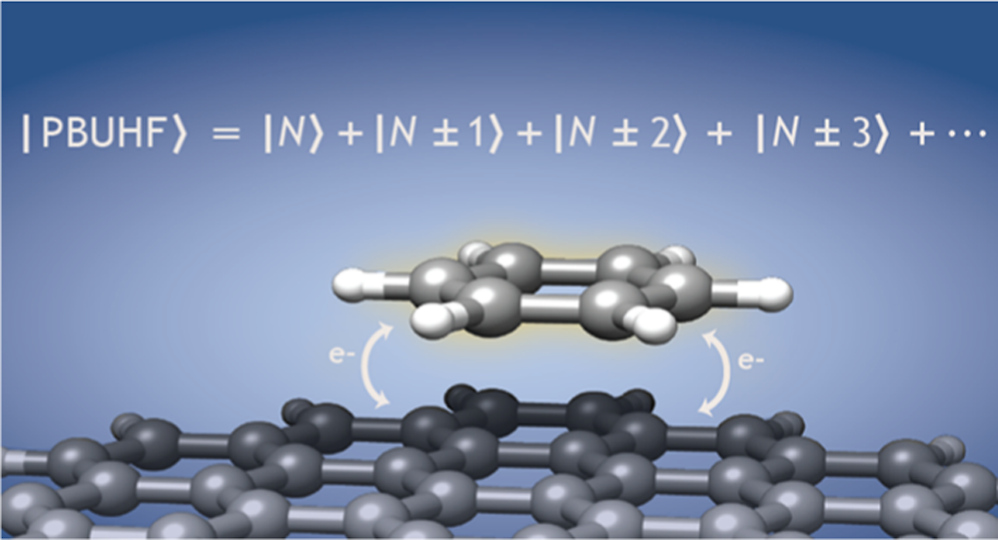

We recently introduced the particle-breaking restricted Hartree–Fock (PBRHF) model, a mean-field approach to address the fractional charging of molecules when they interact with an electronic environment. In this paper, we present an extension of the model referred to as particle-breaking unrestricted Hartree–Fock (PBUHF). The unrestricted formulation contains odd-electron states necessary for a realistic description of fractional charging. Within the PBUHF parametrization, we use two-body operators as they yield convenient operator transformations. However, two-body operators can change only the particle number by two. Therefore, we include noninteracting zero-energy bath orbitals to generate a linear combination of even and odd electron states. Depending on whether the occupied or virtual orbitals of a molecule interact with the environment, the average number of electrons is either decreased or increased. Without interaction, PBUHF reduces to the unrestricted Hartree–Fock wave function.

## 1 Introduction

**Figure 1:**
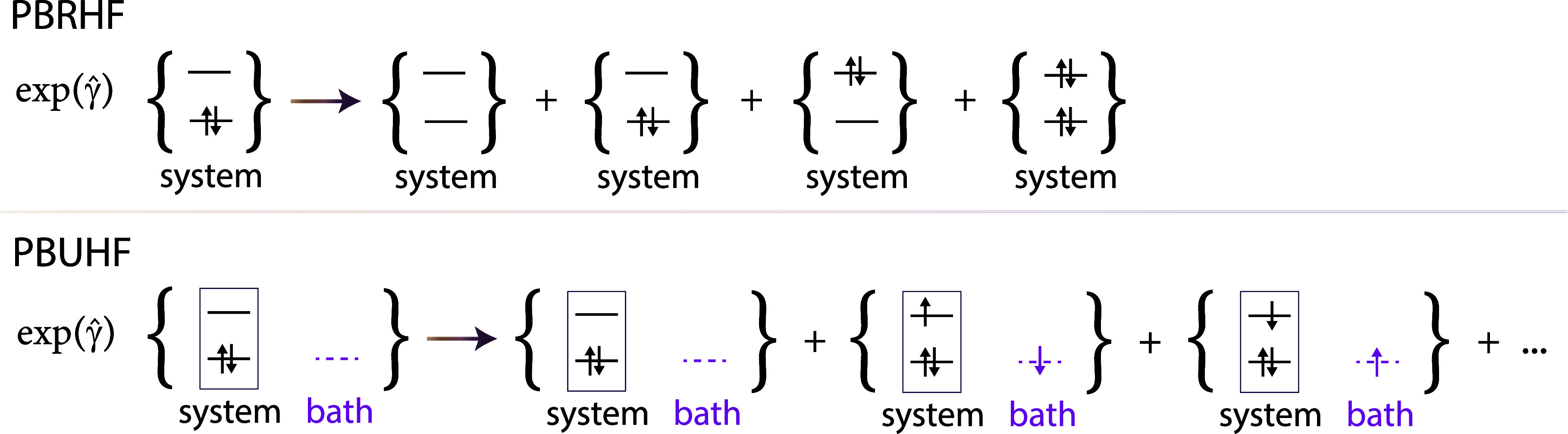
Illustration of the determinants that comprise the PBRHF state (upper panel) compared to the particle-breaking unrestricted Hartree–Fock (PBUHF) state (lower panel) when the same reference is used. When  is applied to a two-electron reference determinant in PBRHF formalism, we obtain a linear combination of determinants containing zero, two, and four electrons. In the case of PBUHF, the bath orbital (empty in this example) provides  flexibility to place one electron in the system and one in the noninteracting bath. Therefore, we get a linear combination of determinants varying by only one electron.

In our recent publication,^1^ we presented the particle-breaking restricted Hartree–Fock (PBRHF) model for molecular systems which are open to electronic charge fluctuations due to interaction with an environment of electronic nature. The target molecule is allowed to be electronically open, so that the effect of the environment on the molecule is taken into account without explicit inclusion of the environment itself. In our formulation, we focus on the particle-breaking nature of the interaction due to charge fluctuations. However, it would also be possible to include charge-conserving interactions as is done in embedding approaches.^2−11^ The particle-breaking nature of interactions may be important for molecules acting as transport junctions,^12,13^ or for molecules that experience effective charging upon interacting with a large or infinite environment.^14,15^ The PBRHF model is a mean-field approach in which the interaction between a molecule and an environment is parametrized through a particle-breaking term in the molecular Hamiltonian. The PBRHF states are linear combinations of closed-shell determinants with *N*, *N* ± 2, *N* ± 4, ... electrons. An example of such a state is shown in the top panel of Figure 1. However, to make the PBRHF approach more flexible for realistic chemical applications, we also need to include odd-electron states in the wave function.

In this paper, we present the PBUHF formalism. Similar to PBRHF, the PBUHF state is generated through a unitary transformation of a reference determinant, parametrized through the exponential of two-body creation and annihilation operators weighted by the occupation parameters. The choice of two-body operators is motivated by the resulting closed-form expressions for the transformed operators. However, the two-body operators can change the number of electrons in the reference state by only even numbers. We include noninteracting orbitals of zero energy^16^ in the orbital space to generate a linear combination of determinants containing both even and odd numbers of electrons. These noninteracting orbitals (hereafter referred to as bath orbitals) have been used for the computation of ionization potentials and electron-attached states in coupled cluster theory,^16−18^ the algebraic diagrammatic construction scheme,^18−20^ and time-dependent density functional theory.^21^ Furthermore, bath orbitals have recently been employed to obtain a spin-adapted coupled cluster model.^22^ In PBUHF, we use bath orbitals to make a subset of two-body operators act as one-electron operators. An illustration in the bottom panel of Figure 1 shows how this works.

The energy of the PBUHF state is minimized with respect to both the occupation parameters and the molecular orbital (MO) coefficients using the trust-region optimization algorithm.^23^ For this purpose, we express the energy in terms of the charge and spin magnetization density matrices. This is analogous to the formulation of the UHF energy by Tsuchimochi et al.,^24^ rather than using the generalized Roothaan’s self-consistent field equations^25^ of Pople and Nesbet,^26^ and Berthier.^27^ Since the PBUHF model can be viewed as a generalization of UHF to electronically open systems, it is beneficial to consider the spin properties of these wave functions. In UHF, α and β electrons are described by different spatial orbitals. Therefore, the UHF wave function is generally not an eigenfunction of the operator for the total spin, . Consequently, the UHF state may be spin contaminated^28^ and many approaches for removing or reducing spin contamination have been developed.^29−33^ The UHF wave function is, however, an eigenfunction of the operator for the projected spin, . In the limit of an electronically closed molecule, PBUHF is equivalent to UHF, and in this case, PBUHF will have the same spin properties as UHF. On the contrary, when a particle-breaking term is included in the Hamiltonian, the PBUHF state will neither be an eigenfunction of  nor . This is a direct consequence of the PBUHF wave function being a linear combination of *N*, *N* ± 1, *N* ± 2, ... electron states. In this paper, we use a particle-breaking Hamiltonian of singlet spin symmetry. In this way, the interaction with the environment induces fractional charging of the molecule without changing its average spin projection. However, since the wave function in this case is not an eigenfunction of , the spread in spin projection is not zero.

The PBHF (PBRHF and PBUHF) model is related to the Hartree–Fock–Bogoliubov (HFB) model,^34^ which has found widespread use in physics.^35−43^ HFB theory unifies the mean-field description with the electron pairing correlation of the Bardeen–Cooper–Schrieffer (BCS) theory^44^ for superconductivity. The HFB wave function breaks the particle-number symmetry by treating the system as a collection of quasiparticles.^45^ These quasiparticles are formally related to geminals, and the HFB wave function connects to the antisymmetrized geminal power wave function^46−50^ through the particle-number projection.^51^ Several flavors of geminal approaches have been developed for use in chemistry.^52^ Both the PBRHF and PBUHF wave functions can be seen as the HFB ground state filled with quasi-particles.

Although the violation of particle number seen in the HFB method is often considered a negative side effect, it has become a strategic approach to capture static correlation within single-reference-based methods in nuclear physics.^53−59^ In molecular electronic structure theory, this strategy has also found application in various HFB- and BCS-related frameworks.^24,60−67^ It is important to note that breaking particle-number conservation is closely connected to fractional orbital occupations. Various examples of their use can be found in density functional theory methods, applied for, e.g., computation of electronic excitations^68,68−72^ and ionization/electron attachment energies,^73−75^ description of bond-breaking processes,^76−81^ and chemical reactivity.^82,83^

The PBHF method targets electronically open molecules for which the Hamiltonian includes an effective environment interaction term that does not commute with the number operator. Consequently, the electronic wave function should not be an eigenstate of the number operator. Hence, the particle-breaking nature of the wave function is not a violation of the particle-number symmetry. PBHF (both restricted and unrestricted versions) may therefore exhibit a fractional average number of electrons. In contrast to correlated *N*-electron models, the fractional orbital occupations in the PBHF model directly result from the wave function being a linear combination of Slater determinants with different numbers of electrons.

The paper is organized as follows. In Section 2 we present the parametrization of the PBUHF wave function, energy expression, and expressions for expectation values of relevant operators. Section 3 contains the computational details. In Section 4, we present results demonstrating that PBUHF reduces to the UHF solution for the standard electronic Hamiltonian together with results for a particle-breaking Hamiltonian. Finally, Section 5 gives a summary and concluding remarks.

## 2 Theory

In this section, we describe the PBUHF formalism. We start by defining the Hamiltonian before moving on to the parametrization of the wave function and the description of how we generate even- and odd-electron states. Then, we give the energy expression and the expectation values of the number operator and spin operators.

### 2.1 Hamiltonian

We consider a Hamiltonian consisting of the standard nonrelativistic molecular electronic Hamiltonian, *H*_mol_, and a particle-breaking term, *H*_pb_ (see ref (1))
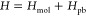
1The particle-breaking Hamiltonian describes an interaction with some environment that causes a spread in the number of electrons. Any particle-conserving interactions with the environment may be included in *H*_mol_, as is done in, e.g., multiscale, embedding, or multilevel approaches, but will not be considered further here. We write the molecular electronic Hamiltonian in terms of the sets of α and β MOs {φ_*p*_^σ^}, where σ = α, β
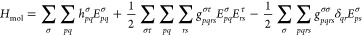
2Here, the nuclear repulsion has been omitted. The summation over σ and τ runs over α and β. The one-electron operator for spin σ in eq 2 is defined as
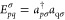
3and the one- and two-electron integrals are, for real MOs, given as
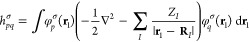
4
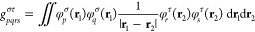
5The integration is over spatial coordinates, *Z*_I_ is the nuclear charge, and ***r***_I_ and ***R***_I_ are the electronic and the nuclear coordinates.

The particle-breaking Hamiltonian
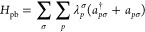
6results from averaging out the environment from an effective one-electron Hamiltonian of the following form
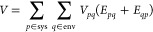
7where *V*_*pq*_ is an electronic coupling strength. The particle-breaking operator in eq 6 does not commute with the number operator for the molecular system, and the parameters λ_*p*_^σ^ (σ = α, β) carry the charge transfer effect of the environment. The choice of the particle-breaking Hamiltonian will be guided by the considerations of the redox properties of both the molecular system and the environment and the strength of the electronic coupling between them.

### 2.2 Parametrization of the Wave Function

We parametrize the PBUHF wave function, |Ψ⟩, in terms of a unitary transformation of a reference determinant, |Φ⟩
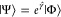
8This is analogous to the PBRHF wave function parametrization. The operator γ̂ is responsible for generating the particle-breaking state, and in the unrestricted case we use
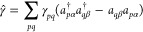
9We note from eq 9 that , such that  is a unitary operator. In this way, the norm of the reference determinant is preserved. Although the two-body operators in eq 9 change the electron number only in multiples of 2 compared to the reference determinant (see upper panel in Figure 1), their use is motivated by the resulting closed-form expressions in eqs 13 and 14. Furthermore, we have chosen γ̂ to be of singlet symmetry (the matrix of the parameters γ_*pq*_ is symmetric and independent of spin), since this simplifies equations considerably. To introduce *N*, *N* ± 1, *N* ± 2, ... determinants in the PBUHF state and access other spin states than that of the reference determinant, we include one or more bath orbitals^16−22^ into the orbital space. When the summation in γ̂ is over the composite orbital space (molecular system + baths), the bath orbitals provide the two-body operators the flexibility to act as one-body operators in the system
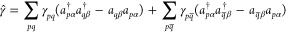
10where unbarred indices refer to system orbitals, and barred indices refer to bath orbitals. For example, a two-body creation operator  adds a single electron to the wave function of the system by simultaneously adding one electron to the bath orbital (see lower panel of Figure 1). Analogously, if the bath is full, a two-body annihilation operator  can remove a single electron from the system. A detailed discussion of the bath setup used in the PBUHF calculations is given in Section 3.

Using bath orbitals, we may write the particle-breaking Hamiltonian given in eq 6 as follows
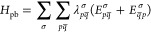
11The summation over *p* is over system orbitals, and the summation over *q̅* includes only bath orbitals. This operator effectively reflects the interaction of the system with its environment, where the parameter  describes the charge transfer effect of the environment. The parameters  are chosen to be spin independent, i.e., . Compared to the PBRHF model,^1^ the particle-breaking Hamiltonian in PBUHF is a one-body creation or annihilation operator in the system and can therefore connect *N* and *N* ± 1 states.

#### 2.2.1 Transformed Operators

We choose to work with operators transformed by  and a reference state, rather than transformed states. Hence, we consider operators transformed according to
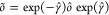
12The expressions for the transformed operators are found using the Baker–Campbell–Hausdorff expansion, and the final expressions for the transformed creation operators are given as
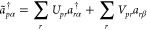
13
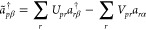
14The transformed annihilation operators can be found by taking the Hermitian adjoints of the creation operators, and all anticommutation relations are preserved. The expansion coefficients in eqs 13 and 14 are given by
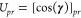
15
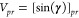
16The elements of **γ** are the parameters in eq 10.

### 2.3 Energy Expression

The electronic energy of the parametrized state |Ψ⟩ is given by the expectation value of the Hamiltonian in eq 1. We minimize the energy simultaneously with respect to orbital rotations and wave function occupation parameters {γ_*pq*_}. The optimization procedure and the relevant equations are described in detail in the Supporting Information.

To obtain the energy expression, we transform the Hamiltonian according to eq 12 and we obtain
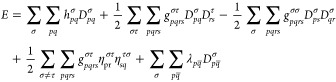
17The first four terms come from *H*_mol_ (eq 2), and the last term comes from *H*_pb_ (eq 11). All summations in the above expression are with respect to system orbitals, except the summation over *q̅*, which is over bath orbitals (see Section 2.2). The standard MO density matrices **D**^σ^ for σ = α, β and the pairing density matrix **η**^στ^ are given by
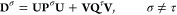
18
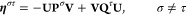
19where the **U** and **V** matrices are symmetric and can be found in eqs 15 and 16. We have introduced the occupied and virtual MO projectors
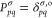
20
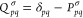
21where δ_*pq*_^σ,o^ = 1 if *p* = *q* and the MO φ_*p*_^σ^ is occupied in the reference determinant, and zero otherwise. We note that the standard MO density matrices are symmetric, whereas the pairing matrices fulfill the symmetry condition .

The optimization is carried out in the MO basis, but the Fock matrices (and similar quantities) are computed in the AO basis. Therefore, the density matrices must be transformed to the AO basis before contraction with two-electron integrals. The AO pairing density matrix is  with σ ≠ τ, and the standard AO σ density matrix is given as
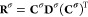
22The standard density matrices fulfill the relaxed idempotence condition^84,85^
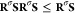
23where the equality only holds for MO densities with integer (one or zero) occupations. I.e., PBUHF density matrices are only idempotent in the limit of a closed molecule (in the case of a standard electronic Hamiltonian) for which the optimized state is a UHF state.

The AO basis is a common basis for both α and β electrons, and this allows us to add and subtract density matrices to simplify the MO energy expression. Following ref (86), we introduce the AO charge density matrix, **P**, and the spin magnetization density matrix, **M**
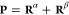
24
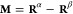
25Using **P** and **M**, we may define the AO Fock matrices, **F**_AO_^α^ and **F**_AO_^β^, as follows
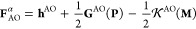
26
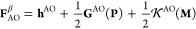
27Here, **h**^AO^ is the one-electron integral matrix in the AO basis, whereas the elements of the two-electron matrices **G**^AO^(**A**) and  are given by
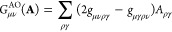
28
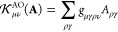
29It can be shown that the Fock matrices in eqs 26 and 27 are identical to the standard UHF Fock matrices.^87^ By transforming the matrices in eqs 26, 27, and 29 to the MO basis, we can write the energy in the MO basis as
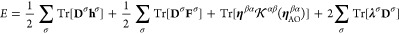
30Note that the MO transform of eq 29 makes use of both α and β MO coefficients through . The MO energy expression in eq 30 is the starting point for the MO-based wave function optimization procedure (Supporting Information).

### 2.4 Expectation Values and Spread of Operators

#### 2.4.1 Electron Spread of the Wave Function

The average number of electrons of the PBUHF wave function is given by the expectation value, . The number operator is given by , where σ = α, β, and in the AO basis we obtain
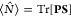
31where **P** is the AO charge density defined in eq 24 and **S** is the AO overlap matrix. The spread in the number of electrons, , is given by
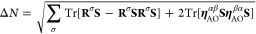
32The AO density matrices **R**^α^ and **R**^β^ are given by the definition in eq 22. From this equation, we can see that only idempotent densities (see eq 23) will have Δ*N* = 0. That is, a PBUHF state can be an electron number eigenstate and thus have a zero electron spread, only for integer occupations (UHF limit), since for integer occupations **R**^σ^**S** – **R**^σ^**SR**^σ^**S** = **0** and **η**^αβ^ = **0**.

#### 2.4.2 Expectation Values of Spin Operators

To evaluate the spin properties of the PBUHF wave function, we compute the expectation values of the operators for the projected, , and total, , spins. In the second quantization, the spin projection operator is given by
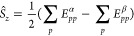
33whereas the operator for total spin is given by . The raising and lowering operators are, in the unrestricted MO basis, defined as  and , where *S*_*pq*_^αβ^ is an element of the αβ MO overlap matrix. The expectation value of  is, in the AO basis, given by
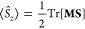
34with a spread in spin projection, , as follows
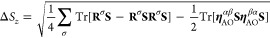
35The spread in Δ*S*_*z*_ will be zero for particle-conserving states as for such states the standard densities are idempotent and the pairing density is zero. This is consistent with the fact that PBUHF reduces to UHF for such states and that UHF is an eigenfunction of the  operator. The expectation value of the  can be calculated in the AO basis as
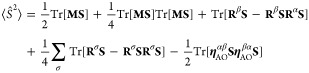
36The last two terms of eq 36 will be zero for particle-conserving UHF states.

## 3 Computational Details

The PBUHF framework is implemented in a development version of the *e*^*T*^ program.^88^ The details of the implementation of the trust-region solver are described in ref (89) based on the work in refs (90−93). For particle-conserving calculations (Section 4.1), a convergence threshold of 10^–9^ on the energy gradient (max gradient norm) is used. For particle-breaking calculations (Section 4.2). we use a convergence threshold of 10^–8^.

### 3.1 Bath Setup

**Figure 2:**
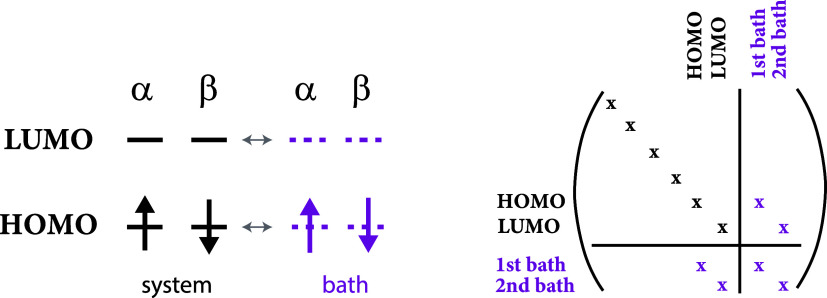
Left panel shows a general bath setup used in PBUHF calculations. In this setup, we connect HOMO and LUMO to two bath orbitals each: one α orbital and one β orbital. The bath orbitals coupled to the HOMO are fully occupied, while the ones connected to the LUMO are empty. In this way, the calculation allows for complete flexibility to remove one electron from the system (from HOMO) and add one electron to the system (LUMO). The right panel illustrates how the bath setup translates to the structure of the **γ** matrix. The crosses indicate nonzero elements.

In this paper, we consider only electron removal/attachment of the highest occupied molecular orbital (HOMO) and the lowest unoccupied molecular orbital (LUMO) of the reference Slater determinant (of eq 8). In the setup, we couple HOMO to a fully occupied bath to give it a flexibility for single electron removal, while LUMO is coupled to an empty bath to allow for the addition of an electron. The bath setup is illustrated in Figure 2. Coupling between bath and system orbitals is introduced through the corresponding elements of parameter matrix **γ** (see eq 10). Additionally, diagonal **γ** elements are allowed to be nonzero. For details, see Section 3.1 in the Supporting Information.

The bath orbitals may be handled easily in the code by augmenting the MO dimension with the number of bath orbitals. The MO coefficients of the bath orbitals in the atomic orbital (AO) basis are zero, as are all integrals involving the baths.

#### 3.1.1 A Special Case: Particle-Conserving Calculations

**Figure 3:**
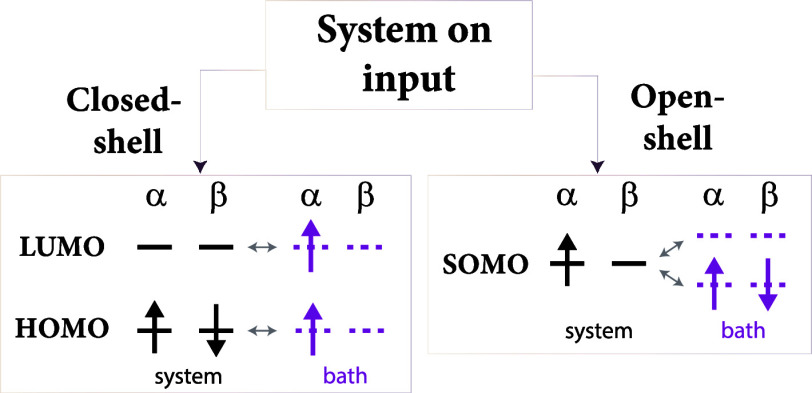
Illustration of the general bath setup used in *particle-conserving* PBUHF calculations. In total, four bath orbitals are used, two α orbitals and two β orbitals. The setup of the baths is different for closed- and open-shell systems on input. If the system is closed shell on input, both HOMO and LUMO are coupled to their own baths with the α orbitals being occupied. If the system is an open shell on input, SOMO is connected to two baths simultaneously. One bath is fully occupied by two electrons of opposite spins, whereas the second bath is empty. Please note that only the aforementioned coupling parameters of **γ** are nonzero.

For the standard molecular electronic Hamiltonian, the PBUHF model can also be used to switch between states with different integer charges. By passing through fractionally charged states, PBUHF can transition from one particle-conserving UHF state to another, given appropriate considerations of spin and spatial symmetries. Since the γ̂ the operator is chosen to be of singlet symmetry, the overall spin symmetry of the composite system is conserved throughout the optimization. The choice of bath occupations depends on whether the molecular system on input is closed- or open-shell; see Figure 3 (and Section 3.2 in Supporting Information for details). Starting with a closed-shell molecule, either HOMO or LUMO can change its occupation, and therefore, they both need to be connected to separate bath orbitals of doublet symmetry (see left side of Figure 3), i.e., the composite system has an overall triplet symmetry. In this way, the calculation has the full flexibility to go up or down in the number of electrons. For molecular systems that are open shells on input, the singly occupied molecular orbital (SOMO) must be connected to both occupied and empty bath orbitals (i.e., overall doublet symmetry) to remove or add an electron from the system (see right side of Figure 3). Since PBUHF can generate states of an equal number of electrons, but of different spatial symmetry, these can be connected by orbital rotations. Therefore, the orbital rotation operator should be projected according to the spatial symmetries of the MOs.

## 4 Results

In this section, we show calculations that demonstrate the features of the PBUHF model. First, we show that the limit for PBUHF for a standard electronic Hamiltonian is a UHF solution and that the number of electrons can be changed from one integer to another during the PBUHF optimization. For one specific example, we demonstrate the seamless transition from one integer state to another by passing through a fractional number of electrons. Second, we show the effect of the particle-breaking Hamiltonian on the properties of the wave function for two chosen strengths of the  parameter.

### 4.1 PBUHF Results for Closed Systems

#### 4.1.1 The UHF Limit

When employing the standard electronic Hamiltonian (eq 2), the PBUHF method applied to closed systems ensures that the optimized state represents the UHF energy minimum with respect to variation in the number of electrons, as well as orbital rotations. To illustrate this, we present two sets of results for the lithium (Li) and beryllium (Be) atoms, water (H_2_O), and oxygen molecules (O_2_) using the cc-pVDZ basis. For one set, the initial molecular charge specified on input is +1, whereas for the other set it is −1. The results are listed in Table 1. For all calculations, initial and final charges are given, as well as initial and final spin projections, . All final PBUHF energies for the particle-conserving systems correspond to the UHF energies for the final charge state and are therefore not explicitly listed.

**Table 1:** PBUHF Optimizations of the Selected Atoms and Molecules in the cc-pVDZ Basis with Initial Molecular Charges of ±1 Using the Standard Electronic Molecular Hamiltonian^a^

	initial		final	
input system	charge	⟨*S*_*z*_⟩	charge	⟨*S*_*z*_⟩
Li^–^	–1	0.0	0	0.5
Li^+^	+1	0.0	0	0.5
Be^–^	–1	0.5	0	0
Be^+^	+1	0.5	0	0
H_2_O^–^	–1	0.5	0	0
H_2_O^+^	+1	0.5	0	0
O_2_^b^	–1	0.5	0	1.0
O_2_^+^^b^	+1	0.5	0	1.0

a Initial and final charges and spin states are presented. The solution for all systems is the UHF state (i.e., Δ*N* = 0 and Δ*S*_*z*_ = 0). We note that for H_2_O and Be the UHF solution is equivalent to the RHF solution.

b We note that for the oxygen molecule the spin symmetry of the composite system is set to be triplet rather than doublet (see Section 3).

As we can see from Table 1, for all calculations with an initial molecular charge ±1 enforced on input, the PBUHF optimizations converge to a corresponding neutral UHF state. In contrast to UHF, PBUHF is able to change the overall atomic/molecular charge if the charge upon starting guess is not a minimum with respect to variation in the number of electrons. During such a PBUHF optimization, the atom/molecule is passing through fractional electron states with nonzero Δ*N* and Δ*S*_*z*_. The final optimized PBUHF state represents a minimum, with respect to both variations in particle number and orbital rotations.

#### 4.1.2 Equivalence with a Grand-Canonical HF Ensemble Formulation

Here, we show that the PBUHF model is equivalent to a grand-canonical HF ensemble (GCHF) formalism^94,95^ for a standard molecular Hamiltonian. The equivalence between the PBUHF wave function and density-operator-based GCHF arises for particle-conserving operators since they cannot connect determinants of different numbers of electrons. We note that the ensemble used in GCHF is a formal device rather than an ensemble representing equilibrium situations in statistical mechanics.^94^

We start with Li^–^ and transition to Li by setting only the γ parameter which connects HOMO to its bath (denoted γ_Hb_) unequal to zero (see Section 2.2)
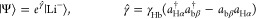
37The γ_Hb_ parameter is chosen on a grid between γ_Hb_ = 0 (Li^–^) and  (Li). For each value of γ_Hb_, we minimize the energy with respect to the orbital rotations.

**Figure 4:**
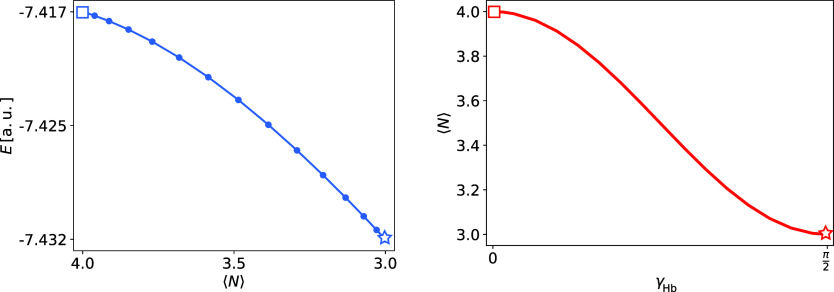
Energies, *E*, (in a.u.) depending on the average number of electrons,  (left panel), and an average number of electrons,  depending on the occupation parameter connecting HOMO and the bath, γ_Hb_, (right panel) for Li atom.

In Figure 4, we plot the obtained energies against the average number of electrons, , (left panel), and the nonlinear dependence of  on γ_Hb_ (right panel). We have indicated the UHF solution for Li^–^ by a square and the UHF solution for Li by a star. Only the points highlighted by the square (Li^–^) and the star (Li) are particle-conserving UHF states. All points in between are fractionally charged states of lithium, and the left panel of Figure 4 displays a continuous and concave energy curve between the two UHF states. However, as expected, the curve will not be continuous at the points representing the Li^–^ and Li states.^96,97^ Hence, for a closed molecule Hamiltonian and fixed γ parameters (not optimized), the PBUHF wave function is related to ensemble HF. However, for a closed system, minimizing the energy with respect to γ parameters will result in a neutral molecule, as can be seen in Section 4.1.1. To understand this, we see from the right panel of Figure 4 that the Li^–^ and Li states represent stationary energy points with respect to γ_Hb_. However, γ_Hb_ = 0 (Li^–^) is a maximum, whereas  (Li) is a minimum.

### 4.2 Results for Particle-Breaking States

To demonstrate the particle-breaking behavior of PBUHF, we consider the hydrogen (H_2_) and water (H_2_O) molecules in the cc-pVDZ basis. We perform three sets of calculations by first coupling HOMO (denoted λ_HOMO_) to its bath orbitals, then LUMO (denoted λ_LUMO_), and finally both HOMO and LUMO. We use the bath setup described in Section 3 and use two coupling strengths:  and . These coupling strengths are chosen to represent strong intermolecular interactions with an environment, based on magnitudes used for hopping integrals of molecules interacting with a surface.^98^ In Table 2, we present average number of electrons, , the electron spread, Δ*N*, energy, *E*, and spin quantities, , Δ*S*_*z*_, and .

**Table 2:** Average Number of Electrons, , Electron Spread, Δ*N*, Energy, *E*, and Spin Properties, , Δ*S*_*z*_, and , from PBUHF Calculations on H_2_ and H_2_O Molecules in the cc-pVDZ Basis for Various λ Combinations^a^

	λ_HOMO_	λ_LUMO_	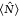	Δ*N*	*E* [a.u.]	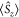	Δ*S*_*z*_	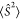
H_2_	0.01	0.0	1.9994	0.0239	–1.129038	0	0.0119	0.0004
	0.0	0.01	2.0051	0.0710	–1.129708	0	0.0355	0.0038
	0.01	0.01	2.0045	0.0750	–1.130047	0	0.0375	0.0042
	0.1	0.0	1.9499	0.2211	–1.161154	0	0.1106	0.0367
	0.0	0.1	2.2386	0.4584	–1.206771	0	0.2292	0.1576
	0.1	0.1	2.1989	0.5421	–1.244104	0	0.2711	0.2204
H_2_O	0.01	0.0	9.9992	0.0290	–75.990207	0	0.0145	0.0006
	0.0	0.01	10.0078	0.0884	–75.991055	0	0.0442	0.0059
	0.01	0.01	10.0070	0.0932	–75.991468	0	0.0466	0.0065
	0.1	0.0	9.9282	0.2631	–76.028835	0	0.1315	0.0519
	0.0	0.1	10.2952	0.5016	–76.079075	0	0.2508	0.1887
	0.1	0.1	10.2405	0.6257	–76.127134	0	0.3129	0.2936

a An isolated neutral H_2_ molecule has 2 electrons, and H_2_O has 10 electrons. The UHF energies for H_2_ and H_2_O in cc-pVDZ basis are −1.128701 a.u. and −75.989796 a.u., respectively.

We first consider calculations for H_2_ and H_2_O when only HOMO is coupled to the environment (λ_LUMO_ = 0). As expected, the average number of electrons decreases in this case. We note that for λ_HOMO_ = 0.01, the average number of electrons is only slightly affected. On the other hand, when only LUMO interacts with the environment (λ_HOMO_ = 0), we observe an increase in . Compared to the results for HOMO interacting with the environment, the effects are more pronounced, even for λ_LUMO_ = 0.01. This is consistent with the fact that ionization is more energy costly than electron attachment for H_2_^99,100^ and H_2_O.^101,102^ Lastly, if both orbitals are involved in the interaction with the same coupling strength (λ_HOMO_ = λ_LUMO_), there is a competing effect between the filling of LUMO and the draining of HOMO, resulting in an overall increase in the average number of electrons. We see that the magnitude of the coupling parameter is decisive for the extent of the induced changes in the average number of electrons and spin properties. As anticipated, the larger the coupling the larger the spread values.

In all presented calculations, the expectation value of the spin projection operator, , is zero. This means that the expected number of α and β electrons in the final particle-broken states is the same. This outcome is to be expected, as there should be no spin polarization as a consequence of the interaction described by *H*_pb_ (see eq 6). The PBUHF state is a mixture of spin states (α and β doublets and the closed-shell singlet) which is seen from the nonzero Δ*S*_*z*_ listed in Table 2. The values of  show that the PBUHF states in Table 2 are predominantly singlet states, but for λ_LUMO_ = 0.1 the admixture of the doublet states becomes pronounced.

## 5 Summary and Concluding Remarks

In this paper, we present the PBUHF model, which can be considered an extension of the previously introduced PBRHF model to open-shell systems. Both PBRHF and PBUHF are mean-field approaches to describe interactions of open molecular systems with an environment of an electronic nature. Such an interaction is given through the so-called particle-breaking term in the Hamiltonian, which contains parameters λ representing electronic coupling strengths and the ability of the molecular system to be reduced or oxidized.

Similarly to PBRHF, PBUHF is based on an exponential unitary transformation of a reference determinant. Since the generator of the unitary operator, γ̂, is derived from two-body creation and annihilation operators, we include noninteracting bath orbitals in the PBUHF optimization. These baths are coupled to the HOMO and LUMO of the system, giving flexibility for the addition or removal of a single electron. In this way, the PBUHF state becomes a linear combination of determinants with both even and odd numbers of electrons. Additionally, γ̂ is of singlet symmetry; i.e., it preserves the spin symmetry of the initial composite system (molecule + baths). In general, the PBUHF wave function is not an eigenfunction of the number operator, *N̂*, or spin projection operator, . However, the average spin projection of the molecule is preserved upon interaction with an environment that has zero spin polarization.

The strength of the coupling in the particle-breaking Hamiltonian determines the extent to which electrons are withdrawn from or added to the molecular system. If the system is interacting with an environment through an occupied orbital, e.g., through HOMO, the electrons will be drained from the system. On the contrary, if an unoccupied orbital, e.g., LUMO, is involved in the interaction, the system will gain electrons.

In the absence of interaction with an environment, i.e., for the standard molecular electronic Hamiltonian, the PBUHF optimization will converge to a UHF solution. In contrast to UHF, if the initial state is not a minimum with respect to the number of electrons, then PBUHF will change it by passing through fractional occupations. The resulting UHF state will be an energy minimum with respect to both orbital rotations and the number of electrons.

## Supporting Information Available

The details on the optimization procedure of the PBUHF wave function and equations for the gradient and the linear transformation of the Hessian on a trial vector and the geometries for the molecules are given in the Supporting Information (PDF)

## Notes

Published as part of *The Journal of Physical Chemistry A* virtual special issue “Roland Lindh Festschrift”.
