# Supplementary material for: Particle-Breaking Unrestricted Hartree–Fock Theory for Open Molecular Systems
Source: J Phys Chem A. 2024 Feb 14;128(8):1533–42. doi: 10.1021/acs.jpca.3c07231 (PMC10910564; doi:10.1021/acs.jpca.3c07231)
Supplement: Supplementary file 1 — jp3c07231_si_001.pdf [file jp3c07231_si_001.pdf]

Supporting Information

Particle-breaking Unrestricted Hartree-Fock Theory for Open Molecular Systems

Regina Paul (nee Matveeva)‡, Sarai Dery Folkestad‡, Bendik Støa Sannes‡, Ida-Marie Høyvik‡*

‡ Dept. of Chemistry, Norwegian University of Science and Technology, 7491 Trondheim, Norway

* E-Mail: Ida-Marie Høyvik – ida-marie.hoyvik@ntnu.no

1 Optimization of the wave function

In this section we present equations needed to optimize the UPBHF wave function using the density matrix based trust-region implementation used for the PBHF wave function. [1, 2] Variations in the wave function are parametrized as,

$$|\Psi\rangle = e^{\hat{\kappa}} e^{\hat{\gamma}+\delta\hat{\gamma}} |\mathrm{R}\rangle \,. \tag{S1}$$

The anti-hermitian orbital-rotation operator $\hat{\kappa}$ for spin-unrestricted rotations is given by, [3]

$$\hat{\kappa}^{\mathrm{UHF}} = \sum_{p>q} \kappa^{\alpha}_{pq}(E^{\alpha}_{pq} - E^{\alpha}_{qp}) + \sum_{p>q} \kappa^{\beta}_{pq}(E^{\beta}_{pq} - E^{\beta}_{qp}), \tag{S2}$$

where E^{σ}_{pq} is the singlet-excitation operator for spin σ and κ^{σ} is an anti-symmetric parameter matrix ($\kappa^{\sigma T} = -\kappa^{\sigma}$). The variations in the standard spin densities are given by

$$\mathbf{D}^{\sigma}(\boldsymbol{\kappa}^{\sigma}, \boldsymbol{\gamma} + \boldsymbol{\delta\gamma}) = \exp(\boldsymbol{\kappa}^{\sigma})\mathbf{D}^{\sigma}(\boldsymbol{\gamma} + \boldsymbol{\delta\gamma}) \exp(-\boldsymbol{\kappa}^{\sigma}), \tag{S3}$$

and the variations of the pairing densities can be expressed as follows

$$\boldsymbol{\eta}^{\sigma\tau}(\boldsymbol{\kappa}^{\sigma}, \boldsymbol{\kappa}^{\tau}, \boldsymbol{\gamma} + \boldsymbol{\delta\gamma}) = \exp(\boldsymbol{\kappa}^{\sigma})\boldsymbol{\eta}^{\sigma\tau}(\boldsymbol{\gamma} + \boldsymbol{\delta\gamma}) \exp(-\boldsymbol{\kappa}^{\tau}). \tag{S4}$$

The definitions of standard, $\mathbf{D}^{\sigma}$, and pairing, $\boldsymbol{\eta}^{\sigma\tau}$, densities are given in Section 2.3 in the main manuscript. The optimization procedure is performed analogously to the optimization described in Ref. 2. Optimization parameters are collected in the vector as follows

$$\mathbf{X} = \begin{pmatrix} \mathrm{vec}(\boldsymbol{\kappa}^{\alpha}) \\ \mathrm{vec}(\boldsymbol{\kappa}^{\beta}) \\ \boldsymbol{\delta\gamma} \end{pmatrix}. \tag{S5}$$

We use the notation introduced in eq S5 to distinguish the parts of the gradient and linear transformation referring to the orbital-rotation parameters and the changes in occupation angles. For the gradient we obtain

$$\mathbf{g}^{\boldsymbol{\kappa}^{\sigma}} = 2[\mathbf{F}^{\sigma}, \mathbf{D}^{\sigma}] + 2\boldsymbol{\mathcal{K}}^{\sigma\tau}(\boldsymbol{\eta}^{\tau\sigma})\boldsymbol{\eta}^{\tau\sigma} - 2\boldsymbol{\eta}^{\sigma\tau}\boldsymbol{\mathcal{K}}^{\tau\sigma}(\boldsymbol{\eta}^{\sigma\tau}), \quad \tau \neq \sigma \tag{S6}$$

$$g^{\gamma}_{pq} = \sum_{\sigma} \mathrm{Tr}[\mathbf{D}^{\sigma,pq}\mathbf{F}^{\sigma}] + \sum_{\sigma\neq\tau} \mathrm{Tr}[\boldsymbol{\mathcal{K}}^{\sigma\tau}(\boldsymbol{\eta}^{\tau\sigma})\boldsymbol{\eta}^{\tau\sigma,pq}] + 2\sum_{\sigma} \mathrm{Tr}[\boldsymbol{\lambda}\mathbf{D}^{\sigma,pq}] \tag{S7}$$

where $\mathbf{F}^{\sigma}$ and $\boldsymbol{\mathcal{K}}^{\sigma\tau}(\boldsymbol{\eta}^{\tau\sigma})$ are defined in Section 2.3 in the main manuscript. In eq (S7) we use the notation

$$\mathbf{D}^{\sigma,pq} = \frac{\partial}{\partial\delta\gamma_{pq}}\mathbf{D}^{\sigma}\Big|_{\boldsymbol{\delta\gamma}=\mathbf{0}} \tag{S8}$$

and likewise for $\boldsymbol{\eta}^{\tau\sigma,pq}$ to denote matrices containing first derivatives of the standard and pairing densities with respect to the change in occupation angles. Elements of $\mathbf{D}^{\sigma,pq}$ and $\boldsymbol{\eta}^{\tau\sigma,pq}$ can be found in Section 2.

For the linear transformation of the Hessian on a trial vector part containing orbital rotation parameters we obtain

$$\boldsymbol{\sigma}^{\boldsymbol{\kappa}^\sigma} = \bar{\boldsymbol{\sigma}}^{\boldsymbol{\kappa}^\sigma} - \bar{\boldsymbol{\sigma}}^{\boldsymbol{\kappa}^\sigma \mathrm{T}}, \tag{S9}$$

where $\bar{\boldsymbol{\sigma}}^{\boldsymbol{\kappa}^\sigma}$ is defined as

$$\begin{aligned}\bar{\boldsymbol{\sigma}}^{\boldsymbol{\kappa}^\sigma} &= -2\mathbf{F}^\sigma[\mathbf{D}^\sigma, \boldsymbol{\kappa}^\sigma] + \mathbf{G}^{\sigma\sigma}(\mathbf{L}_+^{\mathrm{AO}})\mathbf{D}^\sigma - \boldsymbol{\mathcal{K}}^{\sigma\sigma}(\mathbf{L}_-^{\mathrm{AO}})\mathbf{D}^\sigma + 2\sum_{pq}\delta\gamma_{pq}\mathbf{F}^\sigma\mathbf{D}^{\sigma,pq} \\ &+ 2\boldsymbol{\mathcal{K}}^{\sigma\tau}(\boldsymbol{\eta}^{\tau\sigma})\mathbf{R}^{\tau\sigma} + 2\boldsymbol{\mathcal{K}}^{\sigma\tau}(\mathbf{R}^{\tau\sigma})\boldsymbol{\eta}^{\tau\sigma} + \frac{1}{2}\mathbf{g}^{\kappa^\sigma}\boldsymbol{\kappa}^\sigma,\end{aligned} \tag{S10}$$

and the AO matrices, $\mathbf{L}_+^{\mathrm{AO}}$, and $\mathbf{L}_+^{\mathrm{AO}}$ and the MO matrix, $\mathbf{R}^{\tau\sigma}$, are given as follows

$$\mathbf{L}_+^{\mathrm{AO}} = \mathbf{L}_\sigma^{\mathrm{AO}} + \mathbf{L}_\tau^{\mathrm{AO}} \tag{S11}$$

$$\mathbf{L}_-^{\mathrm{AO}} = \mathbf{L}_\sigma^{\mathrm{AO}} - \mathbf{L}_\tau^{\mathrm{AO}} \tag{S12}$$

$$\mathbf{R}^{\tau\sigma} = \boldsymbol{\kappa}^\tau\boldsymbol{\eta}^{\tau\sigma} - \boldsymbol{\eta}^{\tau\sigma}\boldsymbol{\kappa}^\sigma + \sum_{pq}\delta\gamma_{pq}\boldsymbol{\eta}^{\tau\sigma,pq}. \tag{S13}$$

The AO matrix $\mathbf{L}_\sigma^{\mathrm{AO}}$ is defined by the AO transformation of the MO matrix $\mathbf{L}_\sigma$,

$$\mathbf{L}_\sigma = [\boldsymbol{\kappa}^\sigma, \mathbf{D}^\sigma] + \sum_{pq}\delta\gamma_{pq}\mathbf{D}^{\sigma\,pq}. \tag{S14}$$

The linear transformation of the Hessian on a trial vector part containing the changes in occupation angles has elements given by

$$\begin{aligned}\sigma_{tu}^{\gamma} = &-\sum_\sigma \mathrm{Tr}\Big[[\boldsymbol{\kappa}^\sigma, \mathbf{F}^\sigma]\mathbf{D}^{\sigma,tu}\Big] + \frac{1}{2}\sum_\sigma \mathrm{Tr}\Big[\mathbf{G}^{\sigma\sigma}(\mathbf{L}_+^{\mathrm{AO}})\mathbf{D}^{\sigma,tu}\Big] \\ &-\frac{1}{2}\sum_\sigma \mathrm{Tr}\Big[\boldsymbol{\mathcal{K}}^{\sigma\sigma}(\mathbf{L}_-^{\mathrm{AO}})\mathbf{D}^{\sigma,tu}\Big] + \sum_\sigma\sum_{pq}\delta\Gamma_{pq}^\sigma\,\mathrm{Tr}\Big[\mathbf{F}^\sigma\mathbf{D}^{\sigma,tu,pq}\Big] \\ &+ 2\,\mathrm{Tr}\Big[\boldsymbol{\eta}^{\alpha\beta,tu}\boldsymbol{\mathcal{K}}^{\beta\alpha}(\mathbf{R}^{\alpha\beta})\Big] + 2\,\mathrm{Tr}\Big[\mathbf{R}^{\alpha\beta,tu}\boldsymbol{\mathcal{K}}^{\beta\alpha}(\boldsymbol{\eta}^{\alpha\beta})\Big] \\ &+ 2\sum_\sigma \mathrm{Tr}\left[\boldsymbol{\lambda}\sum_{pq}\delta\gamma_{pq}\mathbf{D}^{\sigma,tu,pq}\right]\end{aligned} \tag{S15}$$

where $\mathbf{R}^{\alpha\beta,tu}$ is given by

$$\mathbf{R}^{\alpha\beta,tu} = \boldsymbol{\kappa}^\alpha\boldsymbol{\eta}^{\alpha\beta,tu} - \boldsymbol{\eta}^{\alpha\beta,tu}\boldsymbol{\kappa}^\beta + \sum_{pq}\delta\gamma_{pq}\boldsymbol{\eta}^{\alpha\beta,tu,pq}. \tag{S16}$$

2 Densities differentiated with respect to occupation angles

Here we provide expressions for the elements of the matrices containing first derivatives of the standard and pairing densities with respect to the change in occupation angles:

$$
\begin{aligned}
D_{rs}^{\sigma,pq} &= \sum_{m=1}^{\infty} \frac{(-1)^m}{(2m)!} \sum_{x=0}^{2m-1} \Big([\boldsymbol{\gamma}^x]_{rp}[\mathbf{A}]_{qs} + [\boldsymbol{\gamma}^x]_{rq}[\mathbf{A}]_{ps} + [\mathbf{A}^T]_{rq}[\boldsymbol{\gamma}^x]_{sp} + [\mathbf{A}^T]_{rp}[\boldsymbol{\gamma}^x]_{sq} \\
&- \delta_{pq}\Big([\boldsymbol{\gamma}^x]_{rq}[\mathbf{A}]_{ps} + [\mathbf{A}^T]_{rp}[\boldsymbol{\gamma}^x]_{sq}\Big)\Big) \\
&+ \sum_{m=0}^{\infty} \frac{(-1)^m}{(2m+1)!} \sum_{x=0}^{2m} \Big([\boldsymbol{\gamma}^x]_{rp}[\mathbf{B}]_{qs} + [\boldsymbol{\gamma}^x]_{rq}[\mathbf{B}]_{ps} + [\mathbf{B}^T]_{rq}[\boldsymbol{\gamma}^x]_{sp} + [\mathbf{B}^T]_{rp}[\boldsymbol{\gamma}^x]_{sq} \\
&- \delta_{pq}\Big([\boldsymbol{\gamma}^x]_{rq}[\mathbf{B}]_{ps} + [\mathbf{B}^T]_{rp}[\boldsymbol{\gamma}^x]_{sq}\delta_{pq}\Big)\Big)
\end{aligned}
\tag{S17}
$$

$$
\begin{aligned}
\eta_{rs}^{\tau\sigma,pq} &= \sum_{m=1}^{\infty} \frac{(-1)^m}{(2m)!} \sum_{x=0}^{2m-1} \Big(-[\boldsymbol{\gamma}^x]_{rp}[\mathbf{C}]_{qs} - [\boldsymbol{\gamma}^x]_{rq}[\mathbf{C}]_{ps} + [\boldsymbol{\gamma}^x]_{sp}[\mathbf{D}]_{rq} + [\boldsymbol{\gamma}^x]_{sq}[\mathbf{D}]_{rp} \\
&- \delta_{pq}\Big([\boldsymbol{\gamma}^x]_{sq}[\mathbf{D}]_{rp} - [\boldsymbol{\gamma}^x]_{rq}[\mathbf{C}]_{ps}\Big) \\
&+ \sum_{m=0}^{\infty} \frac{(-1)^m}{(2m+1)!} \sum_{x=0}^{2m} \Big(- [\boldsymbol{\gamma}^x]_{sp}[\mathbf{E}]_{rq} - [\boldsymbol{\gamma}^x]_{sq}[\mathbf{E}]_{rp} + [\boldsymbol{\gamma}^x]_{rp}[\mathbf{F}]_{qs} + [\boldsymbol{\gamma}^x]_{rq}[\mathbf{F}]_{ps} \\
&- \delta_{pq}([\boldsymbol{\gamma}^x]_{rq}[\mathbf{F}]_{ps} - [\boldsymbol{M}]_{sq}^x[\mathbf{E}]_{rp})\Big),
\end{aligned}
\tag{S18}
$$

where $\mathbf{C} = \boldsymbol{\gamma}^{2m-1-x} \cdot \mathbf{P}^\sigma \cdot \Big(\sin[\gamma^x])\Big)^{\mathrm{T}}$, $\mathbf{D} = \sin[\gamma^x] \cdot \mathbf{Q}^\tau \cdot \Big(\gamma^{2m-1-x}\Big)^{\mathrm{T}}$, $\mathbf{E} = \cos[\gamma^x)] \cdot \mathbf{P}^\sigma \cdot \Big(\gamma^{2m-x}\Big)^{\mathrm{T}}$, $\mathbf{F} = \gamma^{2m-x} \cdot \mathbf{Q}^\tau \cdot \Big(\cos[\gamma^x]\Big)^{\mathrm{T}}$.

3 Projections

3.1 Standard calculations

To perform the PBUHF calculations, the only non-zero off-diagonal elements of the matrix γ are parameters coupling the first bath and HOMO, and the second bath and LUMO. Additionally, the diagonal of γ is not zero. The structure of the γ matrix is shown in Figure S1. All other elements of γ are projected out throughout an entire calculation.

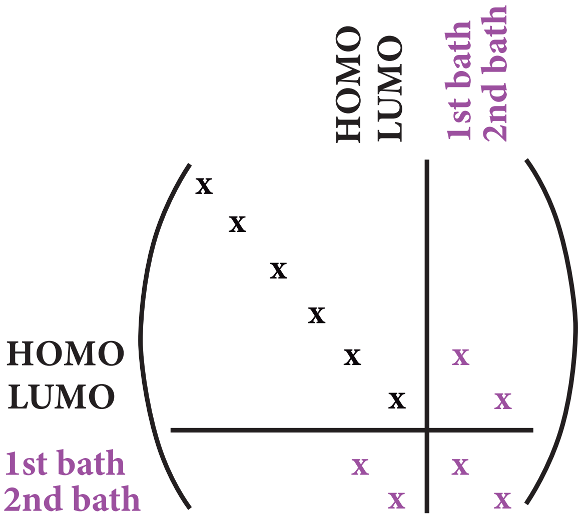

Figure S1: Illustration of the structure of the γ matrix used in PBUHF calculations. Only the diagonal and the off-diagonal elements (marked with "x") coupling HOMO, LUMO and their corresponding baths are not zero.

3.2 Particle-conserving calculations

To perform the *particle-conserving* PBUHF calculations, we distinguish between open- and closed-shell molecular system on input as shown in Figure 3 in the main manuscript. If the system is closed shell on input, the only non-zero elements of the matrix γ are parameters coupling the first bath and HOMO, and the second bath and LUMO. The structure of the γ matrix is shown in Figure S2A. If the system is open shell on input, the only non-zero elements of the matrix γ are parameters coupling the SOMO and the baths. The structure of the γ matrix is shown in Figure S2B. All other elements of γ are projected out throughout an entire calculation. The orbital rotation operator in the construction of the gradient, trial vectors and linear transformation of Hessian on the trial vector should be projected according to the spatial symmetries of the molecular system.

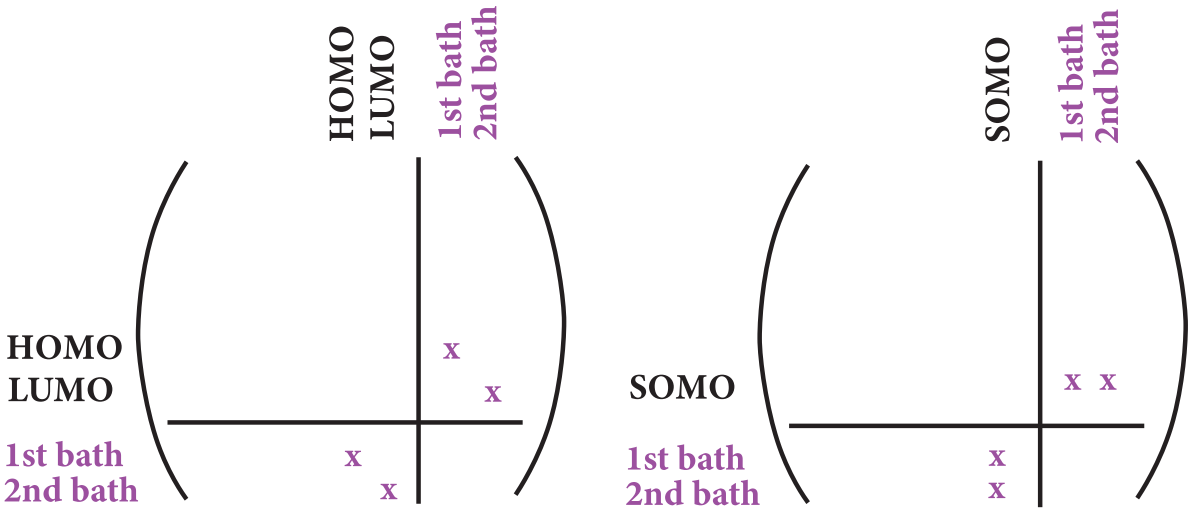

Figure S2: Illustration of the structures of the γ matrix used in *particle-conserving* PBUHF calculations. Left panel. If the molecule is closed-shell on input, only the off-diagonal elements (marked with "x") coupling HOMO, LUMO and their corresponding baths are not zero. Right panel. If the molecule is open-shell on input, only the off-diagonal elements (marked with "x") coupling SOMO and the baths are not zero.

4 Geometries

Geometries for hydrogen, oxygen and water molecules in Ångstrøm.

4.1 H_2

xyz	[Å]		
H	0.00000	0.000000	0.37000
H	0.00000	0.000000	− 0.37000

4.2 O_2

xyz	[Å]		
H	0.00000	0.000000	0.00000
H	0.00000	0.000000	1.20800

4.3 H_2O

xyz	[Å]		
H	0.86681	0.60144	5.00000
H	− 0.86681	0.60144	5.00000
O	0.00000	− 0.07579	5.00000

References

(1) Folkestad, S. D.; Matveeva, R.; Høyvik, I.-M.; Koch, H. *J. Chem. Theory Comput.* **2022**, *18*, 4733–4744.

(2) Matveeva, R.; Folkestad, S. D.; Høyvik, I.-M. *J. Phys. Chem. A* **2023**, *127*, 1329–1341.

(3) Helgaker, T.; Jørgensen, P.; Olsen, J., *Molecular electronic-structure theory*; John Wiley & Sons: 2014.
